# Morphological and proteomic analysis of biofilms from the Antarctic archaeon, *Halorubrum lacusprofundi*

**DOI:** 10.1038/srep37454

**Published:** 2016-11-22

**Authors:** Y. Liao, T. J. Williams, J. Ye, J. Charlesworth, B. P. Burns, A. Poljak, M. J. Raftery, R. Cavicchioli

**Affiliations:** 1 School of Biotechnology and Biomolecular Sciences, The University of New South Wales, Sydney, New South Wales, 2052, Australia; 2 Centre for Marine Bio-Innovation, The University of New South Wales, Sydney, New South Wales, 2052, Australia; 3 Bioanalytical Mass Spectrometry Facility, The University of New South Wales, Sydney, New South Wales, Australia

## Abstract

Biofilms enhance rates of gene exchange, access to specific nutrients, and cell survivability. Haloarchaea in Deep Lake, Antarctica, are characterized by high rates of intergenera gene exchange, metabolic specialization that promotes niche adaptation, and are exposed to high levels of UV-irradiation in summer. *Halorubrum lacusprofundi* from Deep Lake has previously been reported to form biofilms. Here we defined growth conditions that promoted the formation of biofilms and used microscopy and enzymatic digestion of extracellular material to characterize biofilm structures. Extracellular DNA was found to be critical to biofilms, with cell surface proteins and quorum sensing also implicated in biofilm formation. Quantitative proteomics was used to define pathways and cellular processes involved in forming biofilms; these included enhanced purine synthesis and specific cell surface proteins involved in DNA metabolism; post-translational modification of cell surface proteins; specific pathways of carbon metabolism involving acetyl-CoA; and specific responses to oxidative stress. The study provides a new level of understanding about the molecular mechanisms involved in biofilm formation of this important member of the Deep Lake community.

*Halorubrum lacusprofundi* is an important member of Deep Lake in Antarctica, representing ~10% of the lake population^1^. Deep Lake is in the Vestfold Hills, East Antarctica (68°33′36.8S, 78°11′48.7E) and is 36 m deep and perennially cold (down to −20 °C)^1^,^2^,^3^,^4^. The lake was originally a marine environment, having separated from the ocean ~3,500 years ago, and is now a closed system with salinity ~10x marine concentration^1^,^2^,^3^,^4^. Haloarchaea dominate the lake, and a high level of gene exchange occurs throughout the lake’s depth between distinct haloarchaeal genera^1^. The mechanisms of gene exchange have not been determined, although metaproteomic and CRISPR spacer analyses have identified viruses that infect multiple genera, thereby illustrating the potential for gene exchange to occur via transduction of cellular genes^5^. Transformation, conjugation, and cell fusion leading to heterodiploid formation and recombination, have also been considered as potential mechanisms for gene exchange^1^.

By providing high cell-density and cell-cell contact, biofilms may facilitate the exchange of genetic material^6^. Metaproteomics analysis of Deep Lake identified novel pili and cell surface proteins synthesized by the haloarchaea, including *Hrr. lacusprofundi* ACAM34, that were speculated to function in aggregation or attachment^5^. Laboratory studies of *Hrr. lacusprofundi* identified extracellular material and biofilms forming during growth^7^,^8^. The Deep Lake haloarchaea have been shown to possess distinct nutrient preferences, which possibly promotes niche adaptation^1^,^9^,^10^. The Antarctic haloarchaea are also exposed to high levels of UV-irradiation during the Antarctic summer^3^,^4^. Biofilms may therefore not only promote gene exchange, but enhance the survival of haloarchaea to UV-irradiation, and facilitate access to particular types of nutrients; characteristics that have previously been associated with bacterial biofilms^11^,^12^,^13^. Seven Deep Lake isolates (three strains of *Hrr. lacusprofundi* and four strains of *Halohasta litchfieldiae*) were assessed for their ability to adhere to plastic surfaces, and two strains (*Hrr. lacusprofundi* DL28 and *Hht. litchfieldiae* DL24) were found to strongly adhere^8^. The two strongly adhering strains exhibited different biofilm structures, with *Hrr. lacusprofundi* DL28 forming large aggregates and *Hht. litchfieldiae* DL24 forming carpet-like, multilayered biofilms containing macrocolonies. Using staining methods, the biofilms were shown to contain extracellular material consisting of extracellular DNA and glycoconjugates^8^.

Other than these Antarctic haloarchaea, the best characterized biofilm structures for cold-adapted *Archaea* are for SM1 Euryarchaeon that grows in sulfurous marsh waters at ~10 °C^14^,^15^. Forming macroscopic structures (e.g. 3 mm in diameter), the archaeon synthesizes unique appendages (hami) and appears to synthesize a polysaccharide matrix in which it also encases a specific species of *Thiothrix* sipK4 or IMB1 *Epsilonproteobacteria*^15^,^16^,^17^. The biofilm formed is thought to facilitate nutrient exchange between the two species, enabling syntrophic anaerobic sulfur metabolism. In general for *Archaea*, biofilm development has not been well studied, and the composition of extracellular material present in biofilms has been reported to be variable^8^,^18^. The process of cell signaling, or quorum sensing, is often important in biofilm development, but while it has been linked to bacterial biofilms^19^,^20^,^21^, few reports exist for equivalent analyses in *Archaea*; the presence of quorum sensing molecules has been described in haloarchaea^22^,^23^ and a methanogen^24^. A limited number of proteome-based studies have been used to assess archaeal biofilm development, with studies of *Ferroplasma acidarmanus*^25^ and *Sulfolobus* sp.^26^ identifying specific metabolic and morphological characteristics of cells in biofilms.

In view of *Hrr. lacusprofundi* producing extracellular material and forming biofilms, and the potential ecological importance of this capacity, here we used strain ACAM34 to study cell morphology, the composition of extracellular material and quorum sensing associated with biofilms, and used quantitative iTRAQ proteomics to assess the cellular pathways and processes involved. These analyses complement other ongoing studies of this species and collectively serve to expand our understanding of the ecophysiology of cold adapted *Archaea*^27^.

## Results

### Growth conditions leading to biofilm formation

**Figure 1:**
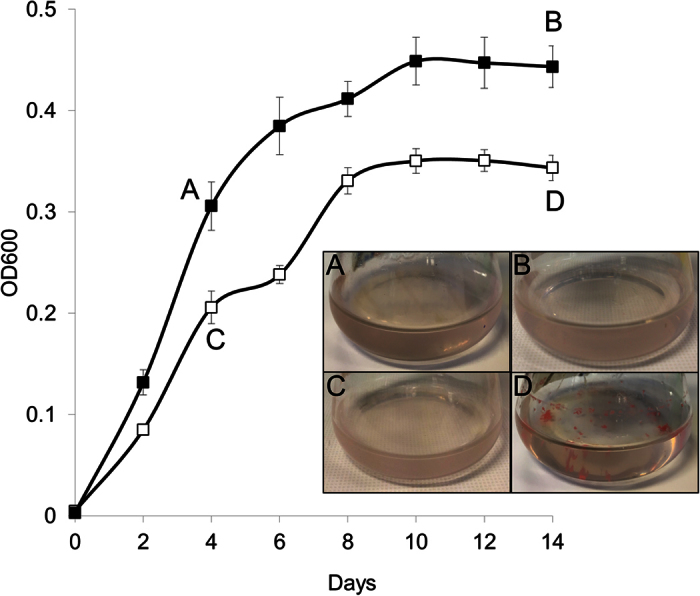
Growth response of *Hrr. lacusprofundi* in medium containing or lacking ammonium. Cultures were grown in 50 mL medium A containing 5 mM NH_4_Cl (full symbols) or medium B lacking NH_4_Cl (open symbols), plus peptone (0.025% w/v) and yeast extract (0.005% w/v) in 250 mL flasks at 30 °C. Error bars represent the standard error of the mean of four experiments. Cells were harvested at various time points for microscopy (see Figs 2 and 3) and proteomics (labelled **A**–**D**). Inset: images of planktonic cells (**A–C**) and biofilms (**D**) in flasks corresponding to the labels on the growth curves.

The ability of media composition to promote biofilm formation of *Hrr. lacusprofundi* ACAM34 was identified during studies aimed at assessing the ability of the strain to utilize urea. *Hrr. lacusprofundi* ACAM34 was previously reported to have weak growth in DBCM2 minimal medium supplemented with low concentrations of peptone (0.025% w/v) and yeast extract (0.005% w/v) plus pyruvate (10 mM) as a carbon source and urea (10 mM) as a nitrogen source^9^. However, the strain was also found to be urease negative with the genome lacking identifiable genes for urea transport or catabolism^9^. To gain further understanding about the capacity of *Hrr. lacusprofundi* to utilize urea, cells were examined throughout the growth phase in medium containing 5 mM NH_4_Cl (medium A) or 5 mM urea (medium B), plus varying concentrations of peptone and yeast extract (Fig. 1, Fig. S1). Growth in the presence of urea, and absence or low concentrations of peptone (2.5 × 10^−4^% w/v) and yeast extract (5 × 10^−5^% w/v), produced essentially no growth over 30 d indicating that urea cannot be used as a sole source of nitrogen by *Hrr. lacusprofundi* ACAM34 (Fig. S1A). In contrast, growth in the presence of urea but with higher concentrations of peptone (0.025% w/v) and yeast extract (0.005% w/v) (i.e. 1 x concentration of medium B) enabled cells to reach OD_600_ > 0.2 (Fig. S1A). When ammonium was substituted for urea (1 x concentration of medium A), a higher growth rate and final OD_600_ (>0.3) was achieved (Fig. S1B), illustrating that, unlike urea, ammonium supports growth of this strain.

These growth studies revealed that the cell aggregation state developed differently in the two types of media. With ammonium added to the medium (medium A), cells remained planktonic throughout the 14 d growth phase, but without the addition of ammonium (medium B), aggregates (floating clumps) formed and then attached to flask walls late in the growth phase (Fig. 1). Aggregates were first visible around day 6, increased in size by day 8, and despite cultures being shaken at 120 rpm, aggregates attached to the walls of the flasks by day 10. The attached aggregates could only be removed by being physically pried off the wall of the flask (e.g. using a wire loop). Between day 10 and 14, planktonic cells, floating aggregates and attached aggregates all remained at similar levels. The floating or attached aggregates are herein referred to as biofilms, based on the definition of biofilms by Costerton and colleagues^28^.

### Cell morphology, extracellular material, and quorum sensing associated with biofilms

**Figure 2:**
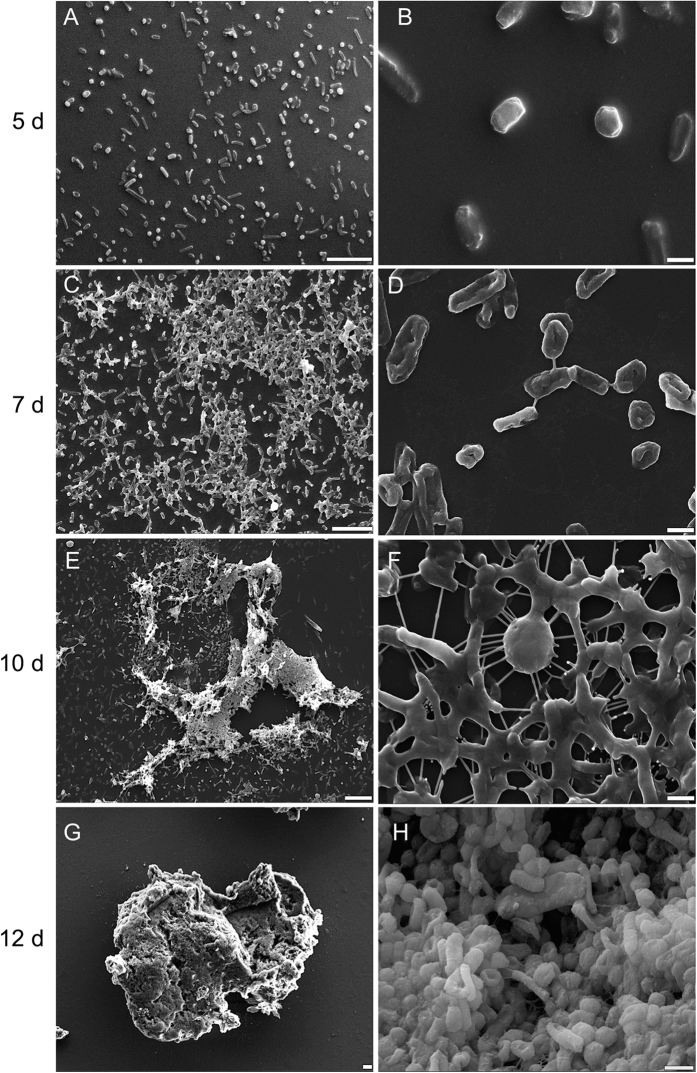
Scanning electron microscopy images of *Hrr. lacusprofundi* biofilm development. Temporal progression (5, 7, 10, 12 d) shown (top to bottom) with images recorded at two different magnifications. (**A,C,E,G**) scale bar, 10 μm; (**B,D,F,H**) scale bar, 1 μm. (**A,B**) Planktonic cells at day 5; individual cells with smooth surfaces. (**C,D**) Cells aggregate and begin to attach day 7; stringy connections visible between cells. (**E,F**) Biofilms grow in size in solution and on the walls of flasks day 10 (images are for cells from biomass scraped of flask walls plus those in solution); protuberances and stringy connections between cells, with cells developing thick layers. (**G,H**) Thick biofilms form day 12–14; day 12 shown; cells with rough surfaces, and large connecting rafts of extracellular material.

To learn about the mechanisms involved in forming biofilms, cell morphology and the presence of extracellular material were assessed using scanning electron microscopy, differential interference contrast microscopy and fluorescence microscopy of cells grown in the absence of added ammonium. Scanning electron microscopy revealed that *Hrr. lacusprofundi* cell shape was pleomorphic, with rods and cocci present (Fig. 2A), consistent with the original description of the organism^29^. Scanning electron microscopy performed on cells from 5, 7, 10 and 12 d revealed an increasing extent of extracellular structures connecting cells (Fig. 2). Cell morphology changed from discrete, relatively smooth cellular units (Fig. 2A,B), to include stringy protuberances (Fig. 2D,F,H) and heavy or rough cell surfaces (Fig. 2H), with sheets or rafts of extracellular material also present (Fig. 2C,E,G).

**Figure 3:**
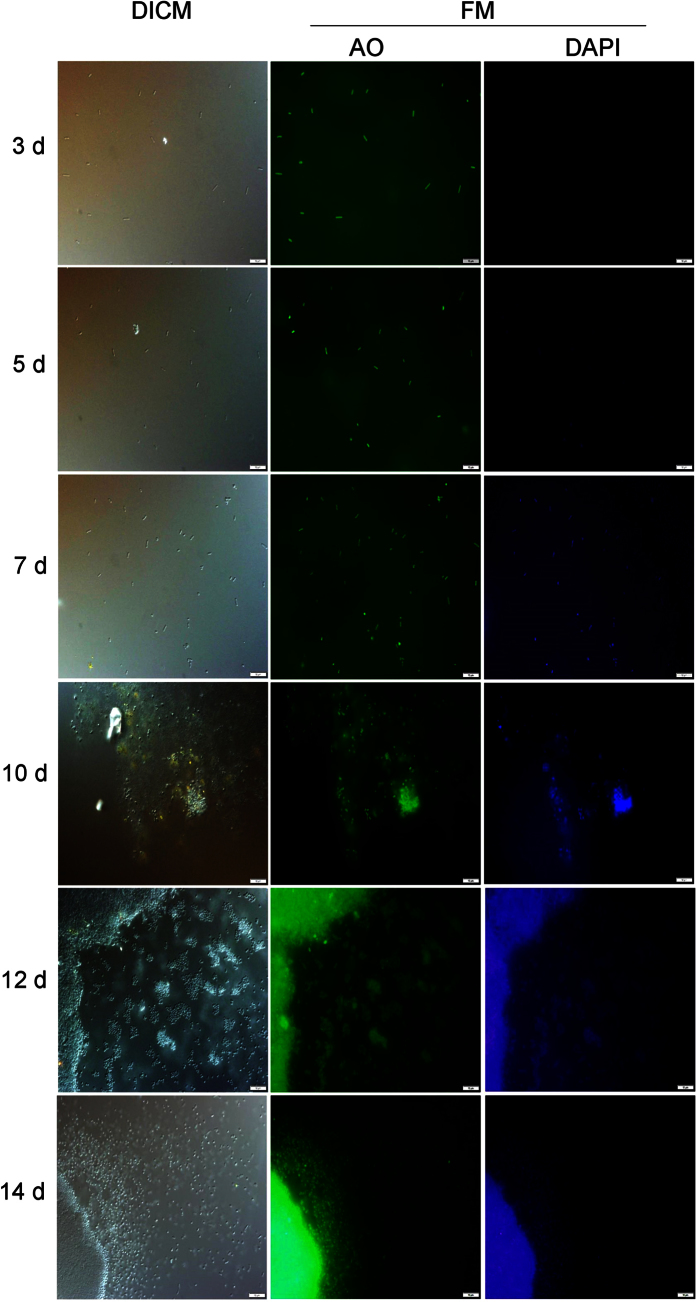
Fluorescence microscopy of *Hrr. lacusprofundi* biofilm development. Images of cells stained with acridine orange (cellular plus extracellular DNA) or 4′,6-diamidino-2-phenylindole (extracellular DNA) compared to differential interference contrast microscopy images. Extracellular DNA in biofilms commenced around day 7 and continued to grow through day 14. The scale bar represents 10 μm. Abbreviations: DICM, differential interference contrast microscopy; FM, fluorescence microscopy; AO, acridine orange; DAPI, 4′,6-diamidino-2-phenylindole.

Cells were stained with acridine orange to highlight cellular and extracellular DNA or 4′,6-diamidino-2-phenylindole to highlight just extracellular DNA^8^,^30^, and fluorescence microscopy images were compared to differential interference contrast microscopy images of unstained cells (Fig. 3). 4′,6-diamidino-2-phenylindole was previously shown to preferentially stain extracellular DNA in haloarchaea and has been used for staining extracellular DNA in haloarchaeal biofilms^8^,^30^. In contrast to planktonic cells (3 and 5 d), 4′,6-diamidino-2-phenylindole signals were higher for biofilm cells (7, 10, 12 and 14 d), indicating that elevated levels of extracellular DNA were present in biofilms (Fig. 3).

**Figure 4:**
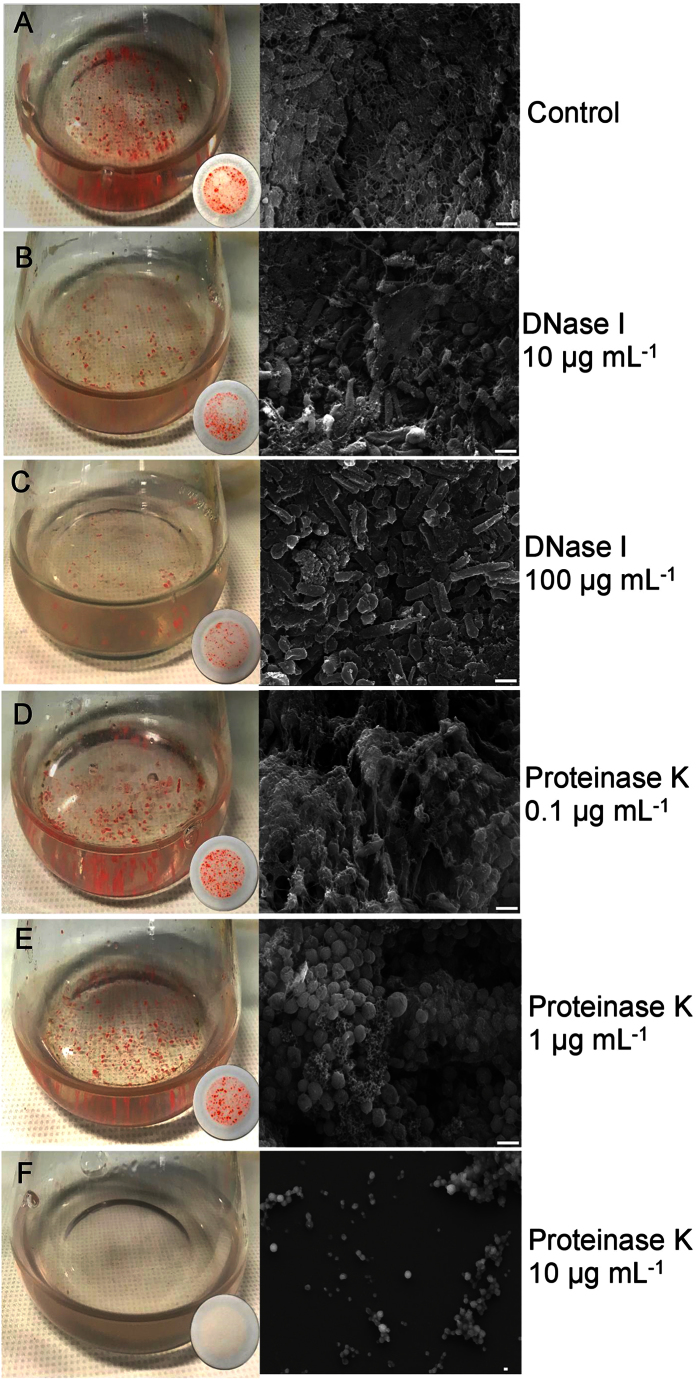
The effects of DNase I or proteinase K on the ability of *Hrr. lacusprofundi* to form biofilms. Cells grown in the absence of DNase I or proteinase K (**A**); 10 μg mL^−1^ DNase I (**B**); 100 μg mL^−1^ DNase I (**C**); 0.1 μg mL^−1^ proteinase K (**D**); 1 μg mL^−1^ proteinase K (**E**); 10 μg mL^−1^ proteinase K (**F**). Biofilms captured on filter papers for each enzyme treatment (25 mL medium, 100 mL flask) are shown beside their respective flasks. The scanning electron microscopy scale bar represents 1 μm. (**A,B,C**) Increasing the concentration of DNase I reduced the extent of extracellular lattice network present between cells. (**D,E,F**) Increasing the concentration of proteinase K led to collapse of the integrity of extracellular material, and changes in cell morphology and number of viable cells.

To further assess the roles of DNA and protein in extracellular material and biofilm formation, cultures were treated with DNase I or proteinase K. DNase I^31^,^32^,^33^ and proteinase K^34^,^35^,^36^ have previously been used to study the structure of biofilms. In order to assess the effects of the enzymatic activity on the formation or disruption of biofilms, the nuclease or protease was added to media before inoculation with cells, or to preformed biofilms, respectively. Quantitation of biofilms was performed by crystal violet staining or bicinchoninic acid assay, with total DNA quantitated by acridine orange staining (Table 1), and cells and extracellular material visualized by scanning electron microscopy (Fig. 4).

**Figure 5:**
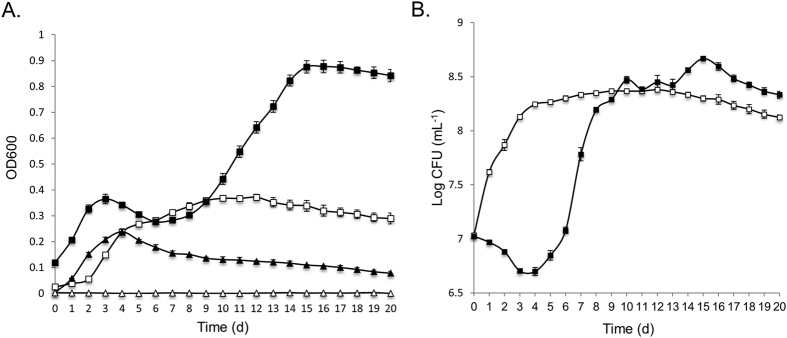
Growth response of *Hrr. lacusprofundi* in medium containing DNase I. *Hrr. lacusprofundi* were grown in 50 mL medium (250 mL flasks) with or without 1 mg mL^−1^ DNase I and OD_600_ (**A**) and viability (**B**) determined. (**A**) OD_600_ for cultures without DNase I (open squares); cultures with 1 mg mL^−1^ DNase I (full squares); uninoculated medium without DNase I (open triangles); uninoculated medium with 1 mg mL^−1^ DNase I (full triangles). (**B**) Colony forming units for cultures without DNase I (open squares); cultures with 1 mg mL^−1^ DNase I (full squares). For uninoculated medium with 1 mg mL^−1^ DNase I, the number of cells per mL was below the detection limit (100 cells mL^−1^) (data not shown). Error bars represent the standard error of the mean of three experiments.

Treatment with DNase I showed a concentration-dependent (0, 10 and 100 μg mL^−1^) reduction in the development of biofilms and total DNA (Table 1, Fig. 4). Scanning electron microscopy revealed a reduction in the extent of lattice-like extracellular material surrounding cells, while the morphology of individual cells remained largely unchanged (Fig. 4). Higher concentrations of DNase I (1 mg mL^−1^) completely inhibited biofilm formation, and led to a large increase in final OD_600_, consistent with the growth of only planktonic cells (Fig. 5A). The high concentration of DNase I (1 mg mL^−1^) produced an early spike in OD_600_ (for cultures and uninoculated controls) (Fig. 5A) consistent with some of the enzyme aggregating in the high salt, and this coincided with a period of no increase in colony forming units (Fig. 5B). However, DNase I activity (assessed by *in vitro* digestion of *Hrr. lacusprofundi* DNA) was retained throughout the 14 d incubation period (Fig. S2). Moreover, the replacement of DNase I with an equivalent concentration of bovine serum albumin (up to 1 mg mL^−1^) led to a spike in OD_600_ (later in the incubation period) (Fig. S3), but had no effect on biofilm formation throughout the 20 d period (data not shown). Collectively the data for DNase I treatments illustrate that the extracellular material contains DNA that is important for biofilm formation.

Proteinase K treatment (0.1 and 1 μg mL^−1^) disrupted lattice-like extracellular material and led to changes in cellular morphology (small spherical cells), with 10 μg mL^−1^ greatly reducing the rate of cell growth, viability, biomass formation and the production of biofilms (Table 1, Fig. 4). Increasing the proteinase K concentration to 100 μg mL^−1^ completely prevented cell growth (data not shown). Similar to DNase I, activity of proteinase K was retained in the growth medium throughout 14 d of incubation (data not shown). While the structure of the extracellular material lost its integrity with relatively low concentrations (0.1 and 1 μg mL^−1^) of proteinase K (Fig. 4), the yield of filtered biofilm biomass and total DNA increased relative to the control (Table 1). These findings are consistent with proteinase K causing the release of DNA from cells, with DNA accumulating in extracellular material and biomass, and all of it being captured on filters.

The biomass of biofilms was unchanged after 10 d-old cultures with preformed biofilms were incubated for a further 4 d with a high concentration of DNase I (1 mg mL^−1^) or proteinase K (10 μg mL^−1^) (data not shown), indicating enzyme activity primarily affected growing cells. The findings are overall consistent with cell growth leading to DNA being released from the cell by active export or cell lysis, with the DNA fulfilling a structural role in the extracellular material involved in biofilm formation. Because proteinase K appeared to cause release of DNA and destroy extracellular material lattice structure, it is likely that cell surface proteins were proteolytically degraded during growth of the cells thereby compromising cell structural integrity and possibly protein structures involved in linkages to extracellular material.

**Figure 6:**
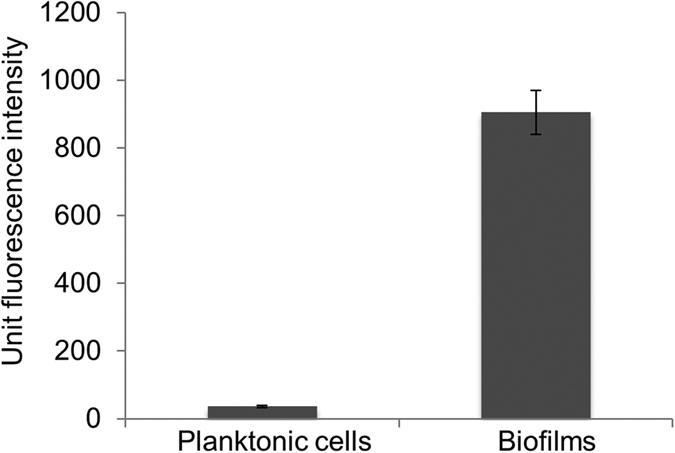
Quorum sensing-like activity of *Hrr. lacusprofundi* associated with biofilm cells. N-acyl homoserine lactone-like quorum sensing was assessed using the supernatant fraction of planktonic cells (4 d growth) or biofilms (14 d growth). Relative fluorescence units from the *E. coli* MT102 biosensor were determined by subtracting the background fluorescence (media blank), and unit fluorescence intensity was shown normalized to protein concentration of whole cell extracts. Error bars represent the standard error of the mean of three experiments. Fluorescence microscopy images of samples are shown in Fig. S4.

The presence of N-acyl homoserine lactone-like quorum sensing molecules was assessed using an *Escherichia coli* green fluorescent protein reporter assay^37^ with the supernatant fraction of planktonic cells (4 d growth) or biofilms (14 d growth) (Fig. 6). Both quantitative measurements that were normalized to protein concentration (Fig. 6) and fluorescence images (Fig. S4) showed markedly higher green fluorescent protein fluorescence in biofilm cells, indicating that N-acyl homoserine lactone-like compounds may be involved in biofilm formation.

### Proteomics of biofilm formation

**Figure 7:**
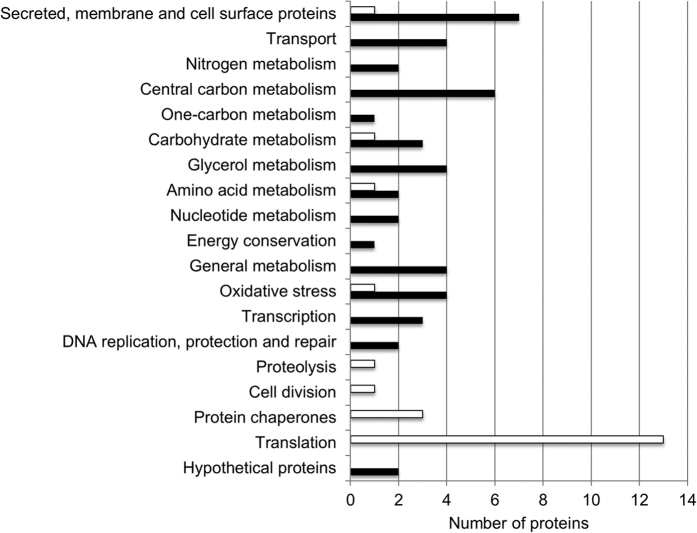
Protein functional categories associated with biofilms or planktonic cells. Proteins were identified using an 8-plex iTRAQ proteomics protocol with higher abundance in biofilms (black bars) or lower abundance in biofilms (white bars) under both the growth phase and growth medium conditions that were tested, and assigned to functional categories (also see Table 2).

Proteomics was performed on whole cells and the extracellular fraction (supernatant) of *Hrr. lacusprofundi* ACAM34 to learn about global gene expression during biofilm formation and from that data infer pathways and cellular processes involved in forming biofilms. A total of 1996 proteins were detected, accounting for ~54% of the 3665 protein-coding genes in *Hrr. lacusprofundi* ACAM34. An 8-plex iTRAQ labelling approach^38^ (Table S1) was used for assessments of differential abundance, enabling multiple growth conditions to be simultaneously compared. A total of 109 proteins had significant abundance differences (≥1.5-fold) between log phase (4 d growth) and stationary phase (14 d growth) in medium lacking ammonium (Table S2); 165 were differentially abundant between stationary phase cultures from media containing or lacking ammonium (Table S2); and 56 proteins were common to both assessments and represented proteins important for biofilms under both the growth phase and growth medium conditions that were tested (Table 2). The core set of 56 proteins included 36 with higher abundance and 20 with lower abundance (Table 2), and described specific cellular processes (Table 2, Fig. 7). A total of 13 additional proteins were added to the core set because they had ≥1.5-fold differential abundance in one assessment (e.g. log vs stationary) and 1.2–1.5 fold differential abundance in the other assessment (e.g. plus or minus ammonium) and were considered relevant to biofilm formation because they belonged to the functional processes already defined by the 56 core proteins (Table 2, Fig. 7). All original iTRAQ datasets for two iTRAQ labelling experiments, which included four biological replicates for each growth condition, are provided in Tables S3–S10.

Proteins associated with biofilms included higher abundances of a nucleoside-diphosphate-sugar epimerase involved in glycosylation (Hlac_1891) and a range of secreted proteins (Hlac_3146, Hlac_1583, Hlac_0389, Hlac_2298, Hlac_2472, Hlac_1867). Specific transporters, and enzymes requiring the imported solutes as cofactors, had higher abundance in biofilms; these included ABC transporter lipoproteins for molybdate (Hlac_2057) and zinc (Hlac_1191), a zinc-dependent formaldehyde dehydrogenase (Hlac_1837), a zinc-dependent carboxypeptidase (Hlac_1583), and a molybdopterin-dependent formate dehydrogenase (Hlac_1238). Consistent with biofilms forming in medium lacking ammonium, proteins with higher abundance related to nitrogen metabolism included an ammonium transporter (Hlac_2623), glutamine synthetase (GS; Hlac_2374), and an acetamidase (Hlac_2285).

Cultures forming biofilms also had higher abundances of metabolic enzymes including: acetyl-CoA synthesis (Hlac_1306, Hlac_0890, Hlac_0891, Hlac_0967); the utilization of acetyl-CoA via the glyoxylate cycle (Hlac_3040; Hlac_2153); the carboxylation of phosphoenolpyruvate (PEP) to oxaloacetate (Hlac_2311); the conversion of glycerol into dihydroxyacetone phosphate (DHAP) (Hlac_2109, Hlac_1458, Hlac_1122, Hlac_1124); and purine synthesis (Hlac_1295, Hlac_1250). Proteins associated with responses to oxidative stress or DNA damage in biofilms included a higher abundance of alkyl hydroperoxide reductase (Ahp; Hlac_1677), a secreted antioxidant protein (YggE homolog; Hlac_2298), the cysteine biosynthesis protein CysK (Hlac_1763), proteins involved in the assembly of iron-sulfur (FeS) clusters (Hlac_0175, Hlac_0176), chromosomal protein MC1 (Hlac_0021), and a predicted DNA helicase (Hlac_3022), and the lower abundance of catalase/peroxidase (HPI; Hlac_1548).

Proteins that decreased in biofilms were mainly involved in protein synthesis and folding, including a large number of ribosomal proteins, and protein chaperones (Table 2, Fig. 7). Additionally, biofilms had lower levels of a proteasome protein (Hlac_0185), a protein translocase (Hlac_2426), and a cell division protein (Hlac_1716) (Table 2).

## Discussion

**Figure 8:**
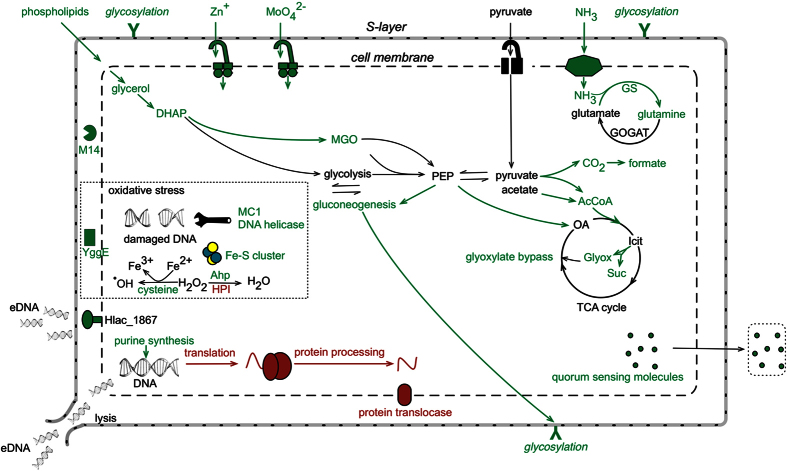
Depiction of the major metabolic pathways and cellular processes in *Hrr. lacusprofundi* involved in forming biofilms. The depiction represents findings for cell morphology, extracellular material, and quorum sensing integrated with the core proteomic data (Fig. 7 and Table 2). Pathways and processes linked to biofilms (green) vs planktonic cells (red) are shown, highlighting the importance of extracellular DNA (including extracellular DNA processing by Hlac_1867 and cellular release by lysis), post-translationally modified cell surface proteins (Y symbol), carbohydrate synthesis, specific responses to oxidative stress, carbon metabolism involving acetyl-CoA, ammonium assimilation and quorum sensing. Abbreviations: eDNA, extracellular DNA; DHAP, dihydroxyacetone phosphate; MGO: methyglyoxal; AcCoA, acetyl-CoA; OA, oxaloacetate; Icit, isocitrate; Glyox, glyoxylate; Suc, succinate; M14, peptidase M14 carboxypeptidase A; YggE, antioxidant protein; MC1, non-histone chromosomal MC1 family protein (Hlac_0021); Ahp, alkyl hydroperoxide reductase; HPI, catalase/peroxidase.

The major metabolic pathways and cellular processes involved in biofilm formation were inferred by integrating the core proteomic data with cell morphology, extracellular material, and quorum sensing data (Fig. 8). The abundance data that were most informative about biofilm formation were the relatively large number of proteins that had elevated levels (see below). However, the proteins that decreased in biofilms, particularly those involved in protein synthesis (Table 2), informed about the overall reduced demand for cell growth; this finding is consistent with bacterial biofilm cells growing more slowly than planktonic cells^39^,^40^,^41^.

### Modification of the cell envelope

In *Archaea*, the surface layer (S-layer) is composed of identical protein subunits arranged into a monolayer, forming a highly porous lattice structure^42^ that maintains the structural integrity of the cell envelope^43^,^44^. Biofilms appear to require post-translational modification of the S-layer. There is evidence for N-glycosylation^44^,^45^ by a nucleoside-diphosphate-sugar epimerase, and peptidolysis by Hlac_1583, which is homologous to an extracellular endopeptidase I from *Lysinibacillus sphaericus* that cleaves spore cortex peptidoglycan^46^,^47^. As Hlac_1583 is a zinc-dependent carboxypeptidase, the elevated levels of the zinc ABC transporter lipoprotein (Hlac_1191) may derive from an increased demand for zinc. The inferred importance of cell envelope proteins is consistent with the potent inhibitory effect of proteinase K on *Hrr. lacusprofundi* biofilm formation (Fig. 4).

### Metabolic networks in biofilm development

Biofilms are associated with changes in carbon metabolism, particularly via acetyl-CoA (Fig. 8). Acetyl-CoA is proposed to function as a metabolic sensor of the availability of nutrients in *E. coli*, and accumulation of acetyl-CoA promotes biofilm production^48^,^49^. The dual action of the glyoxylate cycle (which bypasses the decarboxylation steps of the tricarboxylic acid cycle) and phosphoenolpyruvate carboxylase (which performs anaplerotic fixation of CO_2_) allows conservation of carbon for biosynthetic purposes (such as biosynthesis of carbohydrates). The increased expression of phosphoenolpyruvate carboxylase may relate to sugar synthesis during growth in the media used, which lacks carbohydrates. Phosphoenolpyruvate carboxylase is reported to play a critical role for extracellular material production in biofilm development in *Salmonella*^50^. Thus, it appears that carbon sources that are metabolized to acetyl-CoA and further to specific carbohydrates contribute to biofilm formation in *Hrr. lacusprofundi.*

The increased levels of enzymes involved in glycerol catabolism also appear to represent a route for carbon utilization. However, in this case, cells in biofilms may utilize the glycerophosphate backbones of lipids^51^ from neighboring lysed cells, as has been suggested for phospholipids from *Desulfovibrio vulgaris* biofilms^52^. The data are consistent with evidence of increased cell lysis within biofilms (see eDNA and regulation of biofilm formation). More broadly, these proteomic data highlight the relevance of carbohydrate metabolism in biofilm formation, and may reflect a role for carbohydrates as components of extracellular material involved in forming biofilms.

### Responses to oxidative damage

The increased abundance of a YggE homolog (Hlac_2298) in biofilms was an indicator of oxidative stress as it is homologous to a periplasmic protein from *E. coli* that is inferred to protect cells against oxidative stress^53^,^54^. Intracellularly, cysteine serves as a reductant that drives the Fenton reaction, which generates hydroxyl radicals from iron (II) and hydrogen peroxide, thereby causing damage to DNA^55^. The predicted increase in cysteine biosynthesis (CysK) may therefore enhance oxidative stress. The elevated levels of proteins involved in the assembly of FeS clusters may also be in response to oxidative stress, as has been reported for some bacteria^56^,^57^. Moreover, the increased abundance of a predicted DNA helicase, and chromosomal protein MC1 which has been reported to protect DNA in *Methanosarcina sp.* CHTI 55^58^, may reflect an increased demand to protect and repair DNA damage (Fig. 8).

However, the specific enzymes directly involved in catalyzing the decomposition of hydrogen peroxide indicate that levels of this oxidant may be lower in biofilms than in planktonic cells. Ahp is associated with scavenging low levels of hydrogen peroxide, whereas HPI is associated with high levels^59^. The increase in abundance of Ahp and decrease of HPI in biofilms suggests that under the growth conditions that were used, hydrogen peroxide is present, but at relatively low levels. This would be consistent with biofilms growing more slowly than planktonic cells and thus generating lower levels of hydrogen peroxide. An increased abundance of Ahp was previously identified in biofilms of *Sulfolobus*^26^, several species of *Bacteria*^60^,^61^,^62^, and the fungus *Candida albicans*^63^.

### Extracellular DNA and regulation of biofilm formation

Hlac_1867 is homologous to ComA from *Halobacterium* sp. NRC-1, with both possessing a metallo-*β*-lactamase domain and Lamin Tail Domain (Fig. S5A). In common with Hlac_1867, the *Bacillus subtilis* competence protein ComEC and *Neisseria gonorrhoeae* ComA^64^,^65^ possesses a metallo-*β*-lactamase domain (Fig. S5B), and the *Haloferax volcanii* extracellular DNA metabolism protein Hvo_1477, possesses a Lamin Tail Domain^66^ (Fig. S5A). A Lamin Tail Domain is present in some membrane associated hydrolases and likely affords membrane association^67^. Metallo-*β*-lactamase domains are often associated with nucleases^68^ and have been speculated to directly catalyse hydrolysis of nucleic acids^69^. In Hvo_1477, the metallo-*β*-lactamase domain is replaced by a thermonuclease domain, and the protein has been speculated to function in the *Hfx. volcanii* biofilm lifecycle^66^. Hlac_1867, Hvo_1477 and *Halobacterium* sp. NRC-1 ComA also possess N-terminal lipobox motifs, indicating they may be lipoproteins. The overall functional similarity of these archaeal and bacterial proteins suggests that Hlac_1867 fulfils a role in DNA metabolism in *Hrr. lacusprofundi*, and its elevated abundance in biofilms is consistent with the important role that extracellular DNA plays in biofilm formation (Fig. 3).

The elevated levels of proteins involved in purine biosynthesis may also be a response to the demand for extracellular DNA synthesis during biofilm formation. The requirement for purine synthesis is a characteristic of bacterial biofilms^70^,^71^,^72^; for example, *Bacillus cereus* produces extracellular material containing extracellular DNA, but mutants with defects in purine biosynthesis fail to synthesize biofilms^70^. For some bacterial biofilms, cell lysis and/or secretion of DNA are the main sources of extracellular DNA^73^,^74^,^75^. Both mechanisms seem possible for *Hrr. lacusprofundi*, and if active DNA export does occur, it may be facilitated by Hlac_1867. Given the implications for purine biosynthesis, the role of Hlac_1867, and the effects of DNase I on preventing the formation of biofilms (Figs 4 and 5, Table 1), extracellular DNA stands out as a particularly important component of biofilms in *Hrr. lacusprofundi*.

Cell signaling via N-acyl homoserine lactones has been directly linked to biofilm development, including the processes of initial surface attachment, biofilm maturation and biofilm detachment^20^,^76^. The enhanced fluorescence of quorum-sensing induced biosensors observed with *Hrr. lacusprofundi* biofilms (Figs 6 and S4) suggests quorum sensing may signal biofilm development, perhaps in response to changes in nutrient availability (Fig. 8). The *Hrr. lacusprofundi* genome does not possess genes known to be involved in N-acyl homoserine lactone quorum sensing (e.g. *luxI* or *luxR* homologues), indicating the effector compounds are likely to be mimics of N-acyl homoserine lactones.

### Current knowledge of biofilm development in *Archaea*

A total of three global gene expression studies examining biofilms have been performed on *Archaea*: *F. acidarmanus*^25^, *Sulfolobus* spp.^26^, and *Hrr. lacusprofundi* (this study). The majority of *F. acidarmanus* biofilm associated proteins related to anaerobic growth, consistent with anaerobic zones occurring in some bacterial biofilms^77^,^78^,^79^, and the ability of *F. acidarmanus* to grow anaerobically^25^; a characteristic not shared by *Sulfolobus* spp. or *Hrr. lacusprofundi*. The only class of enzyme with increased abundance from all three studies was Ahp/peroxiredoxin (refs 25, 26 and this study), indicating that a common feature of archaeal biofilms appears to be cells mounting a specific response to relatively low levels of hydrogen peroxide. In *Sulfolobus acidocaldarius* biofilms, an increased abundance of extracellular material, a putative glycosyl transferase and polysaccharides on cell surfaces were reported^26^, which accords with the proposed role of post-translational modification of the S-layer in *Hrr. lacusprofundi* biofilms. In terms of quorum sensing, *Sulfolobus* biofilms were reported to have a decreased abundance of a FabG homolog which is proposed to be involved in the production of a quorum sensing autoinducer^26^, and no evidence for quorum sensing was reported for *F. acidarmanus*^25^; findings that differ from *Hrr. lacusprofundi*. Other than oxidative stress and the role of extracellular material, collectively, these studies do not identify general principles involved in archaeal biofilm formation. This is perhaps not surprising as while the three organisms are members of the *Euryarchaeota*, they represent three very different ecophysiologies: *F. acidarmanus*, iron-dependent chemolithotrophic acidophile^25^; *Sulfolobus* spp., sulfur-dependent heterotrophic/autotrophic thermoacidophile^26^; *Hrr. lacusprofundi*, psychrophilic salt-dependent heterotrophic halophile. Clearly additional types of studies (e.g. gene knockout), and studies of other *Archaea* that form biofilms are warranted.

## Conclusion

By achieving high proteome coverage (~54%), analyzing two cellular fractions (whole cell extract and extracellular fraction) and comparing two distinct growth conditions (with or without ammonium, stationary phase; log vs stationary phase, no ammonium), we obtained strong evidence for specific proteins involved in *Hrr. lacusprofundi* biofilm development. To the best of our knowledge, this is the first quantitative proteomics report to assess biofilms from haloarchaea or Antarctic archaea. The ultrastructural analyses visually characterized the development and extent of extracellular material associated with biofilms, and demonstrated the important role of extracellular DNA in forming biofilms. *Hrr. lacusprofundi* is an important member of the Deep Lake community representing ~10% of the cellular population, and was identified through network analyses as supporting the highest level of intergenera exchange of high identity regions between haloarchaea in the lake^1^. The establishment of proteomic methods for *Hrr. lacusprofundi* coincides well with the development of a system for genetic manipulation of *Hrr. lacusprofundi*^80^, enabling future work to better define the mechanisms involved in formation and regulation of *Hrr. lacusprofundi* biofilms, and the role of biofilms in promoting gene transfer events.

## Methods

### Growth of *Hrr. lacusprofundi*

*Hrr. lacusprofundi* ACAM34 was grown in DBCM2 basal salt medium^81^ supplemented with 10 mM pyruvate and either 5 mM NH_4_Cl (medium A) or 5 mM urea (medium B), plus varying concentrations of peptone and yeast extract (1 x: 5 × 10^−3^% yeast extract and 2.5 × 10^−2^% peptone; 1/10 x: 5 × 10^−4^% yeast extract and 2.5 × 10^−3^% peptone; 1/100 x: 5 × 10^−5^% yeast extract and 2.5 × 10^−4^% peptone; 0 x: no yeast extract or peptone). *Hrr. lacusprofundi* was inoculated 1:100 from cultures grown under the same conditions either in 50 mL medium in 250 mL flasks or 25 mL medium in 100 mL flasks, at 120 rpm. *Hrr. lacusprofundi* grows in the laboratory across a wide range of temperatures (−1 °C to ~44 °C)^7^,^29^ and 30 °C was chosen for cultivation to expedite biomass production and growth assessments, and because the growth regime was suitable for generating both planktonic and biofilm cells; the growth temperature does not simulate natural environmental temperatures in Deep Lake which range from −20 °C at depth to a ~10 °C on the surface in summer^2^,^3^.

### Microscopy and DNase I and Proteinase K treatments

Cells, including biofilms were examined by fluorescence and differential interference contrast microscopy using an Olympus BX61 microscope with DP71 camera (Olympus, Tokyo, Japan) and cellSens Standard 1.11 (Olympus, Tokyo, Japan), and scanning electron microscopy using a JEOL 7001 F field emission scanning electron microscope (JEOL, Freising, Germany), based on previously described methods^8^,^82^. Acridine orange (Ex 505 nm/Em 525 nm) was used at 10 μg mL^−1^ (final concentration) to stain extracellular and intracellular DNA, and 4′,6-diamidino-2-phenylindole (Ex 359 nm/Em 461 nm) was used at 5 μg mL^−1^ (final concentration) to stain extracellular DNA^8^,^30^. To prepare samples for imaging by scanning electron microscopy, 1 mL of cells was directly fixed with 2% glutaraldehyde at 4 °C overnight, cells were washed three times in DBCM2 salt solution to remove glutaraldehyde, and dehydrated sequentially in 30, 50, 70, 90, 100, 100, 100% ethanol, 2:1 ethanol/hexamethyldsilazane, 1:1 ethanol/hexamethyldsilazane, and 100, 100, 100% hexamethyldsilazane for 10 min each. Samples were mounted on scanning electron microscopy sample stubs and chromium coated.

DNase I^31^,^32^,^33^ and proteinase K^34^,^35^,^36^ treatments have previously been used to disrupt biofilms. Here, DNase I (Sigma-Aldrich, MO, USA) or proteinase K from *Tritirachium album* (Sigma-Aldrich) at specific concentrations was added to cultures (25 ml in 100 mL flasks) grown in medium B at the commencement of growth (DNase I: 10 μg mL^−1^, 100 μg mL^−1^, 1 mg mL^−1^; proteinase K: 0.1 μg mL^−1^, 1 μg mL^−1^, 10 μg mL^−1^, 100 μg mL^−1^), or after biofilms had formed (DNase I: 1 mg mL^−1^; proteinase K: 10 μg mL^−1^).

To quantify biofilm biomass, biofilms attached to the inner surfaces of flasks were scraped into solution, and the solution was passed through Whatman no. 54 filter paper using vacuum filtration to collect total biofilms on the filter papers and planktonic cells in the filtrate. The biomass captured on filters was washed three times with a 30% salt water solution^83^. Filter papers were placed in tubes in 25 mL of 30% salt water solution at 30 °C and 120 rpm overnight to release cells into the liquid phase, filters removed, cells pelleted by centrifugation for 5 min at 4,500 × *g* and resuspended in 2 ml 30% salt water solution. For assessments of the retention of DNase I (1 mg mL^−1^) activity in cultures, aliquots (100 μL) were withdrawn daily and incubated with 500 μg *Hrr. lacusprofundi* genomic DNA at 37 °C for 1 h and the sample analysed by agarose (1%) gel electrophoresis. Quantitative assessments of total biofilm biomass using crystal violet (Sigma-Aldrich) was performed based on previously described methods^84^,^85^ and bicinchoninic acid assays were performed using a Thermo Scientific Pierce BCA Protein Assay Kit. The crystal violet method^84^,^85^ detects bacterial peptidoglycan in cell walls, so the method was modified to make it suitable for *Hrr. lacusprofundi*. Additional details of methods are provided in Supplementary Information.

### Quorum sensing

N-acyl homoserine lactone-like compounds were prepared by an ethyl acetate extraction procedure and quorum sensing-like responses determined using an *E. coli* reporter system, based upon published methods^37^,^86^,^87^. *Hrr. lacusprofundi* cells were centrifuged at 4,500 × *g* for 20 min at room temperature and the supernatant recovered. Ethyl acetate extractions^86^ were performed using 200 mL of cell free supernatant and 200 mL of medium B as a control. Extracts were redissolved in 300 μL of methanol acidified with 0.1% (v/v) formic acid, and 10 μL of the redissolved extract and 10 μL of 200 nM oxo-hexanoyl homoserine lactone (Sigma Aldrich) control were evaporated in a 96 well plate (all in triplicate). The reporter strain, *E. coli* MT 102 harbouring the plasmid pJBA132, was grown aerobically in LB broth supplemented with ampicillin (100 μg mL^−1^) at 30 °C with shaking at 150 rpm. Overnight cultures of *E. coli* MT102 (pJBA132) were diluted 10 times in AB minimal medium^88^. A volume of 200 μL of diluted *E. coli* MT102 (pJBA132) was added to each well in the microtiter plate that contained extract, or oxo-hexanoyl homoserine lactone (as a control). Plates were incubated for 5 h at 30 °C at 100 rpm, and GFP fluorescence (485 nm excitation, 535 nm emission) was determined using a CLARIOstar plate reader (BMG Labtech, Ortenberg, Germany) as previously described^37^. Relative fluorescence units were determined by subtracting the background fluorescence (media blank), and fluorescent intensities were normalized to mg of protein present in whole cell extracts. Protein concentration was determined by the bicinchoninic acid method (see Microscopy and DNase I and Proteinase K treatments). Slides for microscopy were prepared using biomass from the wells of the microtitre plates immediately following fluorescence determinations.

### Proteomics

Proteomics was performed based on previously described methods^38^,^89^. Cells were grown in medium A (with ammonium) or medium B (without ammonium) that contained peptone (0.025% w/v) and yeast extract (0.005% w/v). After cells reached mid-logarithmic phase (4 d), half of the culture volume was removed and cells harvested to obtain a whole cell pellet and an extracellular fraction (supernatant). The remaining half of the culture was grown until 14 d, and cells were harvested and processed as for log phase cells. Quantitative proteomics was performed using 8-plex iTRAQ labelling^90^ according to manufacturer’s instructions (Sciex, Framingham, MA, USA) using a specific labelling design (Table S1). In brief, a total of four biological replicates were prepared for each growth condition. The growth conditions were: medium A, log phase (day 4); medium A, stationary phase (day 14); medium B, log phase (day 4); medium B, stationary phase (day 14). For each 8-plex iTRAQ labelling run, two of the biological replicates was used from each growth condition. The approach was used for proteins from whole cell extracts and the extracellular fraction, and two labeling experiments were performed for each fraction. This resulted in a total of four 8-plex iTRAQ labelling experiments and a total of 32 protein samples analyzed. Samples were analyzed using a TripleTOF 5600+ hybrid tandem mass spectrometer (ABSciex, Foster City, USA), and data acquired in information-dependent acquisition mode with Analyst TF 1.7 software (AB Sciex, Foster City, USA). Each 8-plex iTRAQ labelling experiment was run twice to provide two technical replicates. By running technical replicates of each 8-plex iTRAQ labelling experiment, a total of four datasets were generated for whole cell extracts and extracellular fractions of each growth condition. Each of the four datasets for the specific growth condition and fraction were combined and searched with ProteinPilot software 4.5 (AB Sciex, Foster City, USA) against the local *Hrr. lacusprofundi* ACAM34 protein FASTA database to identify proteins. A minimum unused score of 1.3 was accepted for protein identification, representing 95% confidence in correct sequence identification.

For quantitative analysis of relative abundance level changes, data were considered statistically significant when *p* was less than 0.05 and the error factor was less than 2. The weighted average mean and standard deviation of differential abundance between iTRAQ reporter ion ratios were calculated^91^. In addition, an average weighted abundance ratio of 1.5-fold or more was used as the cutoff for differential abundance for assessing proteins involved in biofilm formation. Typically the iTRAQ proteomics literature cites a minimum fold change of 1.2^92^,^93^. In this study, we adopted a more conservative approach in selecting a fold change of 1.5, in addition to a minimum *p* value of 0.05. Pearson’s correlation analysis between biological replicates, technical replicates and labelling experiment replicates was performed using SPSS 22.0 software (Fig. S6). By comparing differential abundance between log phase and stationary phase in medium B, and differential abundance between stationary phase cultures from medium A and medium B, the core set of proteins common to both comparisons represented proteins specific to biofilm development under both the growth phase and growth medium conditions that were tested. In some cases, proteins with 1.2–1.5 fold change were considered if they were from functional categories represented by the core set of proteins (Table 2). The mass spectrometry proteomics data have been deposited to the ProteomeXchange Consortium (http://proteomecentral.proteomexchange.org) via the PRIDE partner repository^94^ with the dataset identifier PXD004202. Additional methodological details are provided in Supplementary Information.

All proteins of relevance were manually functionally annotated based on a previously described approach^95^. InterPro platform (http://www.ebi.ac.uk/interpro/) was used to predict protein domains or signal peptides, and features diagnostic of protein function. Transmembrane domains were predicted by TMHMM Server 2.0 (http://www.cbs.dtu.dk/services/TMHMM/) and annotation as a membrane protein required a positive result from TMHMM. Proteins with N-terminal signal peptides and/or a single C-terminal transmembrane helix and/or homology to experimentally characterized cell surface proteins (e.g. S-layer proteins) were annotated as cell surface/envelope proteins according to a previously described approach^5^.

## Additional Information

**How to cite this article**: Liao, Y. *et al.* Morphological and proteomic analysis of biofilms from the Antarctic archaeon, *Halorubrum lacusprofundi*. *Sci. Rep.* **6**, 37454; doi: 10.1038/srep37454 (2016).

**Publisher's note:** Springer Nature remains neutral with regard to jurisdictional claims in published maps and institutional affiliations.

## Supplementary Material

Supplementary Information

Supplementary Table S2

Supplementary Table S3, S4

Supplementary Table S5, S6

Supplementary Table S7, S8

Supplementary Table S9, S10

## Figures and Tables

**Table 1:** Effect of DNase I and proteinase K on biofilm formation of *Hrr. lacusprofundi*.

Treatment	Biofilm biomass		Total intra- and extra-cellular DNA
	Bicinchoninic acid assay (total protein μg)	Crystal violet assay (OD_600_)	Acridine orange assay [(LAU-B) mm^−2^]
Control	1400 ± 30	1.0 ± 0.1	101000 ± 1100
DNase I 10 μg mL^−1^	1100 ± 60*	0.75 ± 0.08**	76000 ± 710**
DNase I 100 μg mL^−1^	600 ± 40**	0.50 ± 0.05***	26800 ± 270***
Proteinase K 0.1 μg mL^−1^	1900 ± 50*	1.7 ± 0.1***	181000 ± 9700*
Proteinase K 1 μg mL^−1^	1500 ± 60	1.1 ± 0.09	257000 ± 12000**
Proteinase K 10 μg mL^−1^	17 ± 6***	0.03 ± 0.01****	3190 ± 130***

Bicinchoninic acid, crystal violet and acridine orange assays were performed at 10 d. The results are the means ± standard errors from three biological replicates. Asterisks indicate the significance of the difference compared to the value for the control, calculated using a paired t-test: *p < 0.05, **p < 0.01, ***p < 0.001; ****p < 0.0001.

**Table 2:** Proteins from proteomics linked to *Hrr. lacusprofundi* biofilms.

Locus_tag	Annotation	Differential abundance (biofilm vs planktonic)			
		Medium B		Stationary phase	
		Stationary vs log		Medium B vs A	
		WCF	SF	WCF	SF
Secreted, membrane, and cell surface proteins					
**Hlac_0389**	signal peptide; no identifiable domains	ns	1.8	ns	1.8
**Hlac_1583**	signal peptide; peptidase M14 carboxypeptidase A family (zinc-dependent)-possible S-layer modulation	ns	1.6	ns	4.3
**Hlac_1867**	signal peptide; predicted lipoprotein; similarity to bacterial and archaeal DNA (including eDNA) metabolism proteins	—	1.6	—	1.5
*Hlac_2426*	*preprotein translocase, SecY subunit (9 TMDs)*	*0.76*	—	*0.65*	—
Hlac_2472	signal peptide; PGF-CTERM archaeal protein-sorting signal; 1 TMD	ns	1.5	ns	1.3
**Hlac_3146**	TAT signal; no identifiable domains	—	2.1	—	3.2
Transport					
**Hlac_0674**	ABC transporter oligopeptide/dipeptide-binding protein	1.7	ns	2.2	ns
Hlac_1191	ABC transporter zinc-binding lipoprotein	2.0	3.9	1.4	1.3
**Hlac_1631**	electron transport protein SCO1/SenC (signal peptide)	ns	1.8	ns	1.9
**Hlac_2057**	ABC transporter molybdate-binding lipoprotein	0.44	1.6	0.69	1.6
**Hlac_2623**	Rh family protein/ammonium transporter (Amt)	3.3	ns	8.3	ns
Nitrogen metabolism					
**Hlac_2285**	acetamidase/formamidase	3.3	1.6	4.5	2.1
**Hlac_2374**	glutamine synthetase, type I (GlnA)	1.5	2.2	2.7	3.1
Central carbon metabolism					
Hlac_0890	pyruvate ferredoxin: oxidoreductase, beta subunit (PorB)	1.2	ns	1.7	ns
Hlac_0891	pyruvate:ferredoxin oxidoreductase alpha subunit (PorA)	1.2	ns	1.9	ns
**Hlac_1306**	acetate:CoA ligase (Acs)	3.3	ns	2.3	ns
**Hlac_2153**	isocitrate lyase (AceA)	3.9	ns	2.5	ns
**Hlac_2311**	phosphoenolpyruvate carboxylase (Ppc)	1.5	ns	2.0	ns
Hlac_3040	aconitate hydratase	1.3	ns	1.7	ns
One carbon metabolism					
**Hlac_1238**	formate dehydrogenase, alpha subunit (FdhA)	1.5	—	2.0	—
Carbohydrate metabolism					
**Hlac_1300**	gluconate dehydratase (GnaD)	1.6	ns	2.3	ns
***Hlac_1672***	*glyceraldehyde-3-phosphate dehydrogenase, type II (Gap2)*	*0.4*	*ns*	*0.6*	*ns*
**Hlac_1891**	NAD-dependent epimerase/dehydratase (possible glycosylation of S-layer protein)	1.7	ns	1.8	ns
**Hlac_2371**	glyceraldehyde-3-phosphate dehydrogenase, type I (Gap)	1.6	ns	2.2	ns
Glycerol metabolism					
Hlac_1122	glycerol kinase (GlpK)	1.7	ns	1.4	ns
Hlac_1124	glycerol 3-phosphate dehydrogenase subunit B (GlpB)	1.4	—	1.5	—
Hlac_1458	dihydroxyacetone kinase, K subunit (DhaK)	1.3	ns	1.6	ns
**Hlac_2109**	glycerol 2-dehydrogenase (NAD^+^) (GldA)	2.0	—	2.3	—
Amino acid metabolism					
***Hlac_0452***	*phosphoserine phosphatase (SerB)*	*0.46*	*ns*	*0.52*	*ns*
**Hlac_0967**	methylglyoxal synthase	1.5	ns	1.6	ns
Hlac_1941	anthranilate synthase (TrpE)	1.2	—	1.5	—
Nucleotide metabolism					
**Hlac_1250**	phosphoribosylformylglycinamidine synthase (PurS)	1.5	—	1.8	—
**Hlac_1295**	phosphoribosylamine/glycine ligase (PurD)	1.7	ns	2.1	ns
Energy conservation					
**Hlac_2310**	menaquinol–cytochrome-c reductase	ns	1.5	ns	2.0
General metabolism					
**Hlac_0596**	rhodanese domain + metallo-beta-lactamase domain protein	1.5	—	1.6	—
**Hlac_1098**	isochorismatase-like hydrolase	1.6	—	1.8	—
**Hlac_1619**	NAD (P)-binding oxidoreductase domain	1.5	—	1.5	—
**Hlac_1837**	alcohol dehydrogenase, zinc-dependent	2.3	—	2.5	—
Oxidative stress					
Hlac_0175	FeS assembly protein SufB	1.3	ns	1.6	ns
Hlac_0176	FeS assembly ATPase SufC	1.3	ns	1.5	ns
***Hlac_1548***	*catalase/peroxidase (HPI)*	*0.61*	*ns*	*0.50*	*0.50*
**Hlac_1677**	alkyl hydroperoxide reductase (Ahp)/peroxiredoxin	1.5	ns	1.7	ns
**Hlac_1763**	cysteine synthase A (CysK)	1.5	—	1.5	—
**Hlac_2298**	signal peptide; DUF541 domain; homolog of *E. coli* antioxidant protein YggE	—	1.6	—	1.9
Transcription					
**Hlac_0605**	winged helix-turn-helix domain protein	—	1.7	—	1.9
**Hlac_0693**	phosphate uptake regulator, PhoU	1.5	ns	1.6	ns
**Hlac_1558**	putative transcriptional regulator, XRE family	1.5	ns	1.8	ns
DNA replication, protection and repair					
**Hlac_0021**	non-histone chromosomal MC1 family protein	1.5	—	2.1	—
**Hlac_3022**	type III restriction protein Res subunit	1.5	—	1.5	—
Proteolysis					
***Hlac_0185***	*proteasome regulatory subunit (PsmR)*	*0.61*	*ns*	*0.67*	*ns*
Cell division					
*Hlac_1716*	*cell division control protein CdcH*	*0.71*	*ns*	*0.62*	*ns*
Protein chaperones					
***Hlac_0416***	*group II chaperonin*	*0.45*	*0.63*	*0.61*	*0.70*
***Hlac_0682***	*chaperone protein DnaK*	*0.65*	*ns*	*0.51*	*ns*
***Hlac_2662***	*group II chaperonin*	*0.42*	*ns*	*0.36*	*ns*
Translation					
***Hlac_0100***	*ribosomal protein S7*	*0.66*	*ns*	*0.67*	*ns*
***Hlac_0413***	*ribosomal protein L15e*	*0.67*	*ns*	*0.65*	*ns*
***Hlac_0615***	*ribosomal S13S15 domain protein*	*0.58*	*ns*	*0.62*	*ns*
***Hlac_0618***	*ribosomal protein S3Ae*	*0.63*	*ns*	*0.59*	*ns*
***Hlac_0827***	*ribosomal protein L31e*	*0.61*	*ns*	*0.47*	*ns*
***Hlac_1816***	*ribosomal protein S13*	*0.59*	*ns*	*0.59*	*ns*
***Hlac_1842***	*ribosomal protein L7Ae/L30e/S12e/Gadd45*	*0.62*	*ns*	*0.63*	*ns*
***Hlac_2277***	*ribosomal protein L21e*	*0.47*	—	*0.67*	—
***Hlac_2312***	*ribosomal protein S19e*	*0.63*	*ns*	*0.67*	*ns*
***Hlac_2436***	*ribosomal protein L5*	*0.65*	*ns*	*0.63*	*ns*
***Hlac_2447***	*ribosomal protein L25/L23*	*0.67*	*0.69*	*0.66*	*ns*
***Hlac_2448***	*ribosomal protein L4/L1e*	*0.66*	*ns*	*0.63*	*ns*
***Hlac_2534***	*ribosomal protein L10*	*0.61*	*ns*	*0.61*	*ns*
Uncategorized proteins					
**Hlac_0492**	no identifiable domains	1.8	ns	1.7	ns
**Hlac_1516**	DUF555 domain; uncharacterised protein family UPF0212	1.8	ns	1.7	ns

Proteins associated with biofilms (higher differential abundance, normal font) or planktonic cells (lower differential abundance, italic font) under both the growth phase and growth medium conditions that were tested. Protein functions assigned based on manual annotation. Differential abundance values are expressed as ratios for two fractions: whole cell fraction (WCF) or extracellular supernatant fraction (SF). Core proteins (total of 56) with significant differential abundance (≥1.5-fold) in both assessments (medium B, stationary vs log phase; stationary phase, medium B vs medium A) are shown in bold font; proteins (total of 13) from functional categories represented by core proteins with ≥1.5-fold differential abundance in one assessment and 1.2–0.5 fold differential abundance in the other assessment are shown in plain font. Abbreviations: predicted transmembrane domain, TMD; twin-arginine translocation signal, TAT signal; detected in the expressed proteome, but not with significant differential abundance, ns; not detected in the expressed proteome, -.
