# Supplementary material for: Morphological and proteomic analysis of biofilms from the Antarctic archaeon, Halorubrum lacusprofundi
Source: Sci Rep. 2016 Nov 22;6:37454. doi: 10.1038/srep37454 (PMC5118699; doi:10.1038/srep37454)
Supplement: Supplementary Information [file srep37454-s1.pdf]

Morphological and proteomic analysis of biofilms from the Antarctic archaeon, *Halorubrum lacusprofundi*

Liao Y, Williams TJ, Ye J, Charlesworth J, Burns BP, Poljak A, Raftery MJ and Cavicchioli R

Supplementary Information

Methods

Reference

Figure S1-S6

Table S1-S10 (note Table S2-S10 are provided as separate excel files)

Methods

Preparing samples for iTRAQ labeling. *Hrr. lacusprofundi* ACAM34 cells were grown in DBCM2 basal salt medium [1] supplemented with 10 mM pyruvate and either 5 mM NH_4Cl (medium A) or 5 mM urea (medium B), plus peptone (0.025 % w/v) and yeast extract (0.005% w/v). *Hrr. lacusprofundi* was inoculated 1:100 from cultures grown under the same conditions in 50 mL medium in 250 mL flasks at 30°C and 120 rpm. When cells reached mid-logarithmic phase (4 d), half of the culture volume was removed and cells harvested to obtain a whole cell pellet and an extracellular fraction (supernatant). The remaining half of the culture was grown until 14 d, and cells were harvested and processed as for log phase cells. Cells were pelleted by centrifugation for 20 min at 4,500 x *g*. The supernatant was obtained by filtering cells through a 15 mL Amicon centrifugal concentration unit (Millipore, Billerica, MA) with a 3 kDa cutoff by centrifugation at 5000 × *g*, with five subsequent buffer exchanges with 25 mM $NaHCO_3$. The resulting concentrate (~ 500 µL) constituted the supernatant fraction, and was stored at -80°C until needed. Proteins were extracted by vortexing cell pellets in 5 mL disruption buffer (50 mM Tris-HCl pH 7.2; 2 mM EDTA, pH 7.2; 0.4 mM phenylmethanesulphonylfluoride) and ultrasonically disrupting cells on ice using a Branson Sonifier 250 (Branson Ultrasonics, Danbury, CT) with the probe output set at 20% amplitude for five periods of 40 s (pulse cycle of 0.5 s on and 0.5 s off) with a 40 s cooling on ice between periods of sonication to prevent sample heating which might denature proteins and create solubility problems. The resulting solution was retained and filtered, as described above for the supernatant fraction, to generate the whole cell extract fraction. The protein concentration of each sample in whole cell extracts and supernatant fractions by performing a bicinchoninic acid assay at 562 nm with a microplate reader using a Thermo Scientific Pierce BCA Protein Assay Kit (Product No. 23225) and following manufacturer's instructions. Protein quality was assessed using polyacrylamide-gel electrophoresis (NuPAGE 4-12% Bis-Tris).

iTRAQ labelling. Prior to iTRAQ labelling, protein samples were reduced, alkylated and digested with trypsin overnight. Protein from whole cell extracts or supernatant fractions (100 µg) was reduced with 2 µL tris-(2-carboxyethyl) phosphine and incubated at 60°C for 60 min, and proteins were then alkylated with 1 µL

iodoacetamide (37 mg mL^{-1}) for 10 min at room temperature and then digested with 2 μg trypsin per 100 μg total protein overnight (16 – 18 h) at 37°C. Proteins were labelled with iTRAQ reporter labels according to the manufacturer's instructions (Sciex, Framingham, MA, USA), and the pH adjusted to 8-9 by adding 2.5 - 4 μL of 500 mM Na_2CO_3, followed by incubation for 2 h at room temperature. iTRAQ labelled peptides were combined and then diluted 10-fold with cation exchange "load buffer" (10 mM KH_2PO_4 in 25% acetonitrile, at pH 3.0) to reduce the concentration of buffer salts and organics, and the pH adjusted to 2.5 with 100% acetic acid. The sample mixture was passed slowly through a cation exchange cartridge (AB Sciex, Foster City, CA), and the cartridge was washed with "load buffer" to remove excess reagents (e.g. iTRAQ labels, trypsin, SDS, solvents). The peptides bonding to the cartridge were eluted with 500 μL of cation exchange "elute" buffer (10 mM potassium phosphate KH_2PO_4 in 25% acetonitrile and 350 mM KCl, pH 3.0). The eluent was dried in a speedvac and then resuspended in 500 μL 0.2% heptafluorobutyric acid. A C18 macrotrap (Michrom Bioresources, Auburn, AL), was primed with 1 mL 100% CH_3CN, then 1 mL 50% CH_3CN/0.1% formic, and finally equilibrated with 1 mL 0.2% heptafluorobutyric acid. The 500 μL of sample eluent from the previous step was then loaded into the primed C18 cartridge, and washed with 1 mL 0.2% heptafluorobutyric acid. In order to maximize recovery, the above C18 flow through was passed through an Oasis HLB cartridge (Waters, Milford, MA) to capture any peptides not bound to the macrotrap. The Oasis HLB cartridge was prepared by washing with 3 x 1 mL 100% CH_3CN, then 3 x 1mL 50% CH_3CN/0.1% formic, followed by 3 x 1 mL 0.2% heptafluorobutyric acid. The peptides bound to the C18 cartridge were eluted with 500 μL 50% CH_3CN/0.1% formic, followed by 200 μL 100% CH_3CN. The peptide eluents (C18 and Oasis HLB) were pooled and then dried in a speedvac. The pellets were resuspended in 200 μL of 0.2% heptafluorobutyric acid /0.1% formic acid, and used for LCMSMS analysis.

LC Packings capillary HPLC system. Appropriate dilutions of peptide suspensions (~ 4 μg) were analysed using a nanoLC system consisting of a Dionex UltiMate 3000 RSLCnano pump system, Switchos valve unit and Famos autosampler. Samples were injected onto a C18 precolumn cartridge (Acclaim PepMap 100, 5 mm 100 Å, Thermo Scientific Dionex, Waltham, USA), washed (10 min), and then switched

inline to a capillary column (10 cm, C18 reverse phase packing material; Reprosil-Pur, 1.9 μ, 200 Å, Dr. Maisch GmbH, Ammerbuch-Entringen, Germany). Elution was achieved using a 240 min gradient of buffer A (H_2O:CH_3CN of 98:2 containing 0.1% formic acid) to buffer B (H_2O:CH_3CN of 20:80 containing 0.1% formic acid) at 200 nL min^{-1}.

Mass spectrometry. Using a TripleTOF 5600+ hybrid tandem mass spectrometer (ABSciex, Foster City, USA), ion spray voltage (2300 V) was applied to the Nanospray® III ion source near the column outlet, with fixed angle spraying (25°), and the column tip positioned ~1 cm from the TripleTOF 5600+ source orifice. The interface heater temperature was set to 150°C, positive ion electrospray ionization was used, and the TripleTOF 5600+ operated in information-dependent acquisition mode. A time of flight MS survey scan was acquired (*m/z* 400-1600, 0.4 s) and up to 10 multiply charged ions (*m/z* 400-1250, counts > 400, charge state 2^+, 3^+, 4^+ or 5^+) sequentially selected by Q1 for MS-MS analysis. Nitrogen collision gas was used and optimum collision energy automatically chosen (based on charge state and mass). Tandem mass spectra were accumulated for 0.3 s (*m/z* 100-1800).

Data analysis. The parameters for Protein pilot software 4.5 were set as follows: sample type-iTRAQ 8plex (peptide labeled); Cys alkylation-iodoacetamide; digestion-trypsin; Instrument-TripleTOF 5600; special Factors-none; species-none; ID focus-biological modifications; search effort-thorough ID; detected Protein Threshold-0.05 (10.0%); quantitate and bias correction were applied. False Discovery Rate Analysis was performed. All protein identifications were based on the criteria of False Discovery Rate < 1%. Proteins identified with confidence ≥ 95% (Unused score ≥ 1.3) were taken into account, while the proteins identified without iTRAQ reporter ion ratio data or *p* value were filtered out.

Quantification of biofilm biomass. To quantify biofilm biomass, biofilms attached to the inner surfaces of flasks were scraped into solution, and the solution was passed through Whatman no. 54 filter paper using vacuum filtration to collect total biofilm on the filter paper and planktonic cells in the filtrate. The biomass captured on filters was washed 3 times with a 30% salt water solution[1]. Filter papers were placed in

tubes in 25 mL of 30% salt water solution at 30°C and shaken at 120 rpm overnight to release cells into the liquid phase, filters removed, cells pelleted by centrifugation for 5 min at 4,500 x *g* and cells resuspended in 2 ml 30% salt water solution and stored at room temperature. Prior to analysis, cells were pelleted by centrifugation, and supernatant solution removed as described above. Pellets were air dried and stained with 2 mL 0.1% crystal violet (in 30% salt water solution) for 1-2 h at room temperature. A small amount of cell suspension (1 μL) was examined by light microscopy every 20 min to assess color development. When all the cell pellets turned purple, the excess crystal violet solution was removed by centrifugation at 10,000 x *g* for 2 min, and the cell pellet washed with 3 x 30% salt water solution and then air dried. The dye bound to the cells was resolubilized with 1 mL of 33% glacial acetic acid and the cells kept at room temperature without shaking until the cell pellets changed to their original pink color (1-2 h). The concentration of crystal violet was determined by measuring the optical density of the destaining solution at 600 nm. To quantify biofilm biomass using the bicinchoninic acid assay, total protein was extracted by sonication and protein concentration measured as described above (**iTRAQ labeling**). Acridine orange (Ex 505 nm/Em 525 nm) staining of extracellular and intracellular DNA present in biofilms was performed by suspending biofilms in 2 mL 30% salt water solution and transferring the suspension to 24-well microtitre plates supplemented with acridine orange (final concentration of 10 μg mL^{-1}) and incubating the plates for 15 min in the dark at room temperature. The fluorescence signal was quantified using a Fujifilm FLA-5000 Fluorescent Image Analyzer (Fujifilm, Tokyo, Japan) with a 473 nm excitation laser and Fujifilm LPB filter using Fujifilm Science Lab Image Gauge Ver 4.0 software. Salt water solution (30%) was used as a blank and assessments were performed in triplicate, and standard error calculated.

References

1. Dyall-Smith, M. *The Halohandbook: Protocols for Halobacterial Genetics*. (2009) http://www.haloarchaea.com/resources/handbook/Halohandbook_2009_v7.1.pdf.
2. Dehal, P.S., *et al.* MicrobesOnline: an integrated portal for comparative and functional genomics. *Nucleic Acids Res.* **38,** 396–400 (2010).

3. Narasimhan, G. *et al.* Mining protein sequences for motifs. *J. Comput. Biol.* **9,** 707–720 (2002).

Figure S1. Growth of *Hrr. lacusprofundi* in medium containing or lacking ammonium, with different concentrations of yeast extract and peptone. A: Growth of *Hrr. lacusprofundi* in 25 mL medium B (lacking NH_4Cl) in 100 mL flasks at 30°C. B: Growth of *Hrr. lacusprofundi* in 25 mL medium A (containing 5 mM NH_4Cl) in 100 mL flasks at 30°C. A and B: The growth media contained different concentrations of yeast extract (YE) and peptone (P). 1 x: 5 x 10^{-3}% YE and 2.5 x 10^{-2} % P (purple); 1/10 x: 5 x 10^{-4} % YE and 2.5 x10^{-3} % P (green); 1/100 x: 5 x 10^{-5} % YE and 2.5 x 10^{-4} % P (red); 0 x: no YE or P (blue). Images showing the cultures in their flasks at the end of the incubation periods are shown
under the scatter plots.

Figure S1

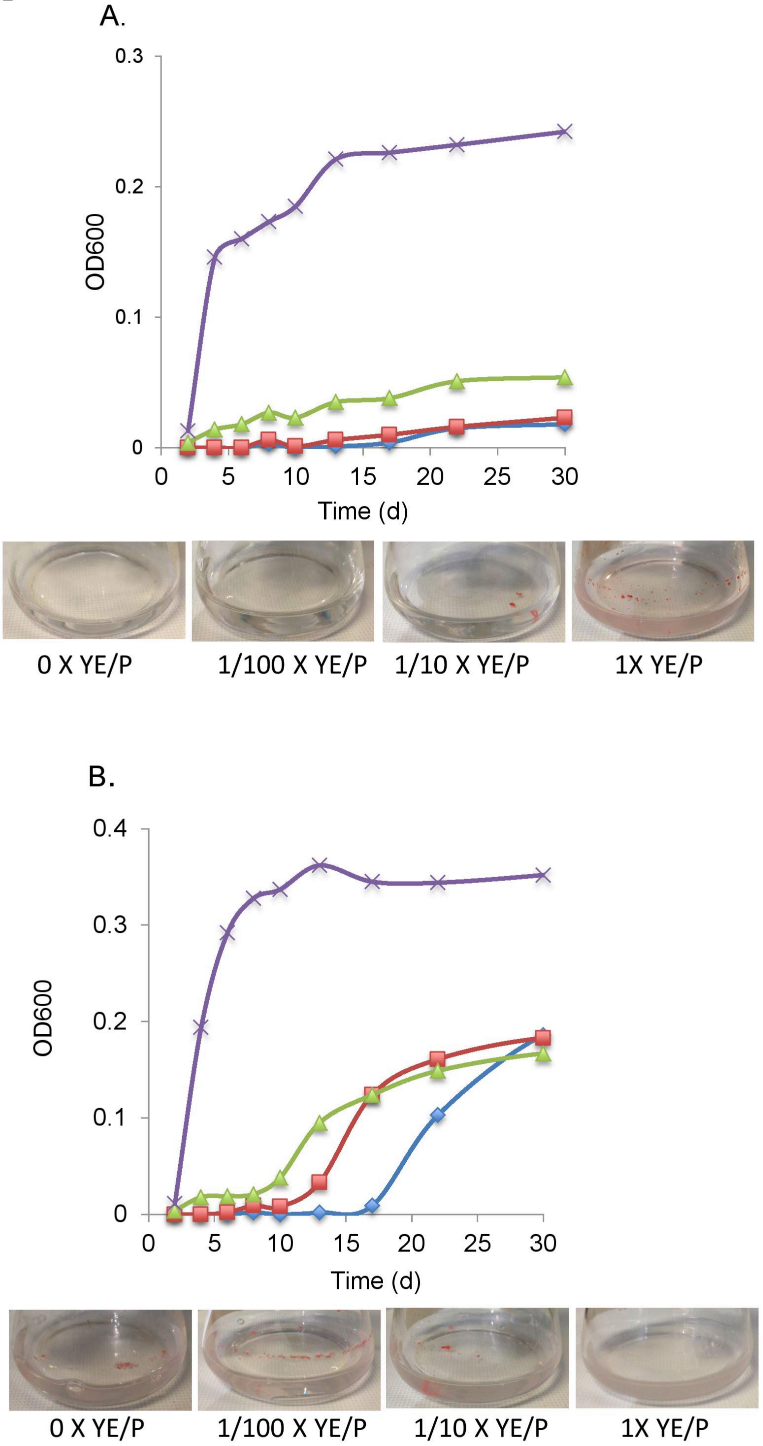
A.
0.3
0.2
0.1
0
OD600
0
5
10
15
20
25
30
Time (d)
0 X YE/P
1/100 X YE/P
1/10 X YE/P
1X YE/P
B.
0.4
0.3
0.2
0.1
0
OD600
0
5
10
15
20
25
30
Time (d)
0 X YE/P
1/100 X YE/P
1/10 X YE/P
1X YE/P

Figure S2. Retention of DNase I activity in incubated cultures. Aliquots (100 µL) were withdrawn daily for 14 d from DNase I-treated cultures (1 mg mL^{-1}) and a DNase I-treated uninoculated control (medium B), and incubated with 500 µg *Hrr. lacusprofundi* genomic DNA at 37°C for 1 h, and the sample analysed by agarose (1%) gel electrophoresis. Lane M: 1 kb DNA ladder; Lane 1: 500 µg haloarchaea genomic DNA in pure water; Lane 2: 500 µg haloarchaea genomic DNA in uninoculated medium B; Lane B: DNase I-treated uninoculated medium B + 500 µg genomic DNA; Lane C: DNase I-treated cultures + 500 µg genomic DNA; Lane W: DNase I-treated pure water + 500 µg genomic DNA.

Figure S2

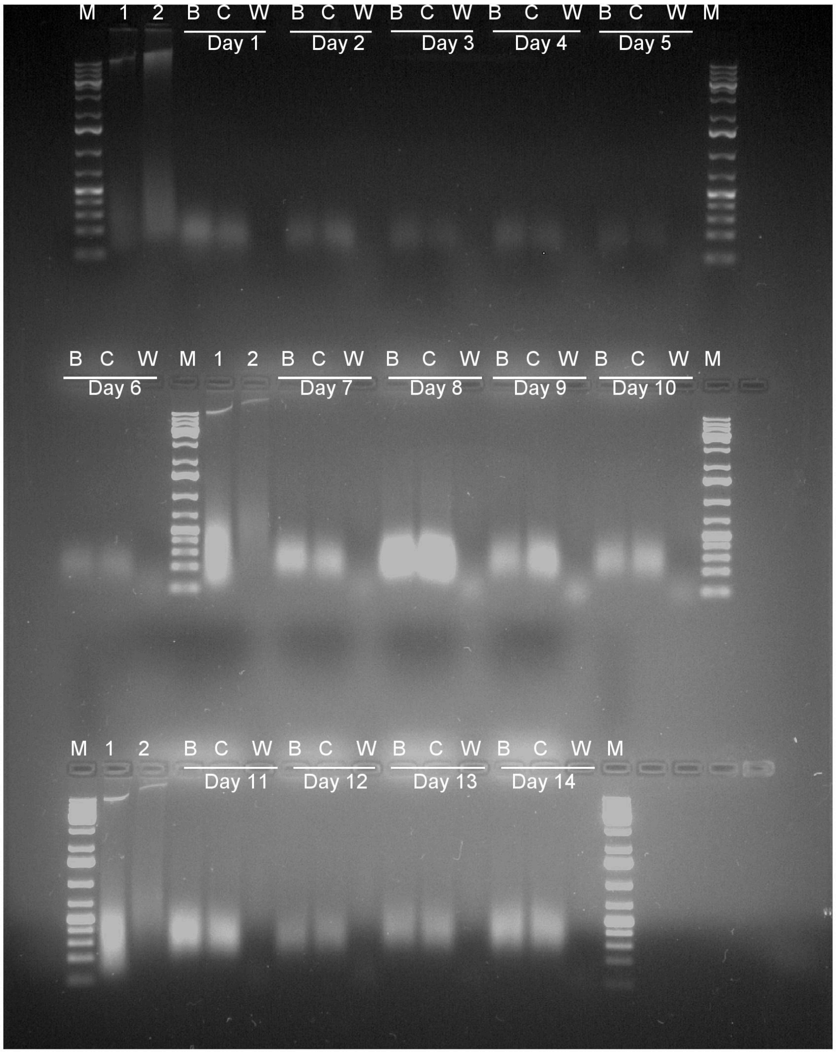
M 1 2 B C W B C W B C W B C W B C W M
Day 1 Day 2 Day 3 Day 4 Day 5
B C W M 1 2 B C W B C W B C W B C W M
Day 6 Day 7 Day 8 Day 9 Day 10
M 1 2 B C W B C W B C W B C W M
Day 11 Day 12 Day 13 Day 14

Figure S3. Growth of *Hrr. lacsurpfundi* in medium lacking ammonium with bovine serum albumin treatment. Growth of *Hrr. lacusprofundi* in 50 mL medium B in 250 mL flasks with the addition of 1 mg mL^{-1} bovine serum albumin. Error bars represent the standard error of the mean for three experiments. A: OD_{600} for cultures without bovine serum albumin (open squares); cultures with 1 mg mL^{-1} bovine serum albumin (full squares); uninoculated medium without bovine serum albumin (open triangles); uninoculated medium with 1 mg mL^{-1} bovine serum albumin (full triangles). B: Cell viability (colony forming units) for cultures without bovine serum albumin (open squares); cultures with 1 mg mL^{-1} bovine serum albumin (full squares); For uninoculated medium with 1 mg mL^{-1} bovine serum albumin, the number of cells per mL was below the detection limit (100 cells mL^{-1}) (data not shown).

Figure S3

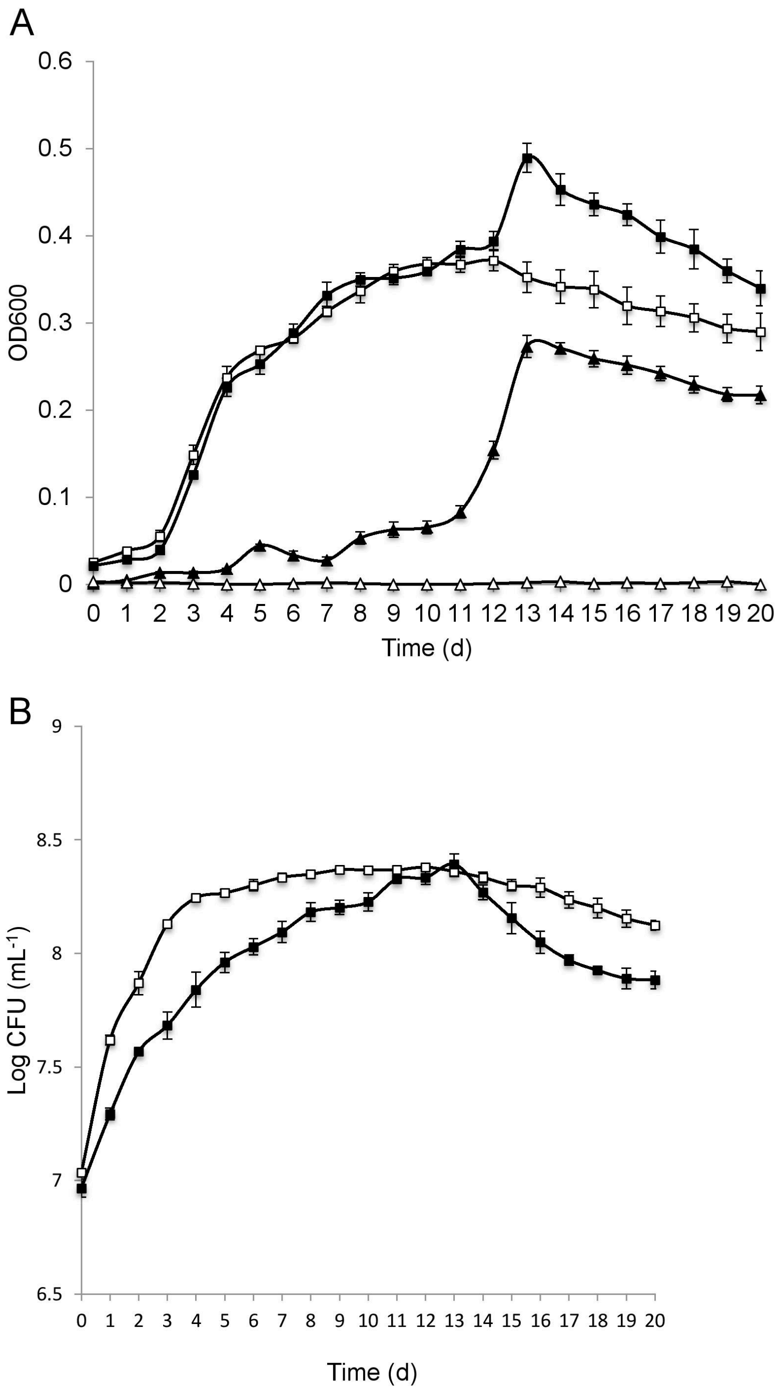

A
0.6
0.5
0.4
0.3
0.2
0.1
0
OD600
0 1 2 3 4 5 6 7 8 9 10 11 12 13 14 15 16 17 18 19 20
Time (d)
B
9
8.5
8
7.5
7
6.5
Log CFU (mL-1)
0 1 2 3 4 5 6 7 8 9 10 11 12 13 14 15 16 17 18 19 20
Time (d)

Figure S4. Quorum sensing of *Hrr. lacusprofundi* associated with biofilm cells. N-acyl homoserine lactone-like quorum sensing was assessed using the supernatant fraction of planktonic cells (4 d growth) or biofilms (14 d growth), as for Fig. 6, and fluorescence microscopy images were recorded. Uninoculated medium B was used as negative control. The scale bar represents 100 μm.

Figure S4

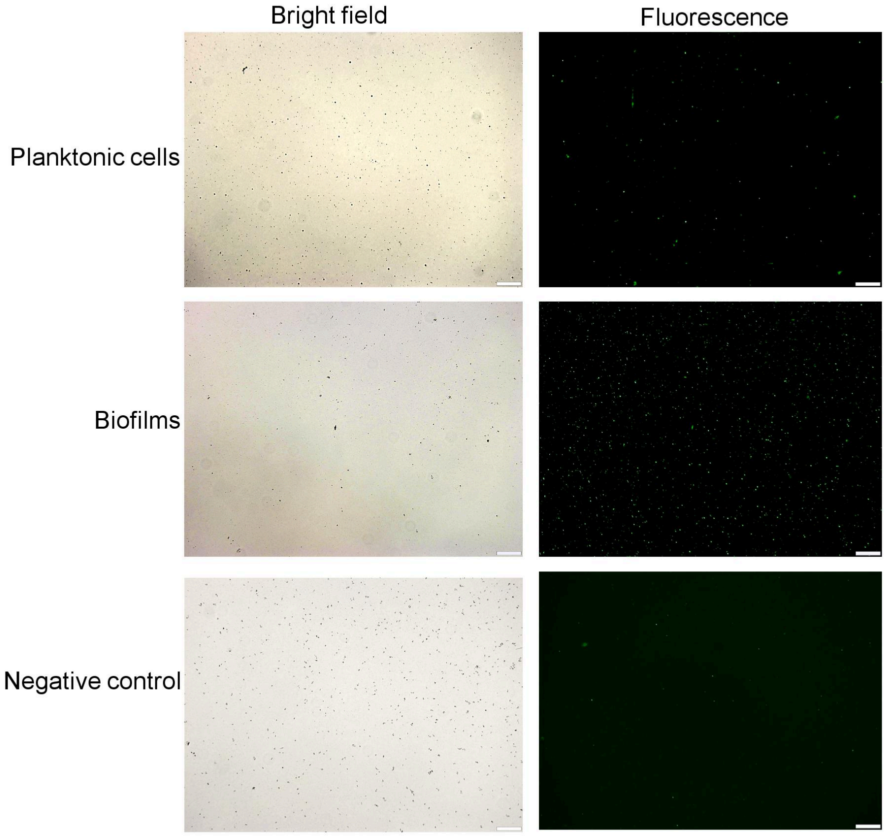

Bright field
Fluorescence
Planktonic cells
Biofilms
Negative control

Figure S5. Protein sequence and domain similarity of *Hrr. lacusprofundi* Hlac_1867 and other archaeal and bacterial DNA metabolism proteins.

A: Hlac_1867 (ACM57445.1) compared to *Hfx. volcanii* Hvo_1477 (WP_004043458.1) and *Halobacterium* sp. NRC-1 ComA (WP_010904188.1).

B: Hlac_1867 compared to *B. subtilis* ComEC (WP_009967776.1) and *N. gonorrhoeae* ComA (KLR91205.1). A and B: Percent amino acid sequence identity determined by BLAST (grey with amino acid numbers and % identity); Domain structures: COGs protein domains and protein superfamilies identified using the MicrobesOnLine portal[2]; Lipobox motif (lipo) predicted by searching against interPro platform; Helix-turn-helix motifs (HTH) predicted using GYM 2.0 (http://users.cis.fiu.edu/~giri/bioinf/GYM2/welcome.html)[3].

Figure S5

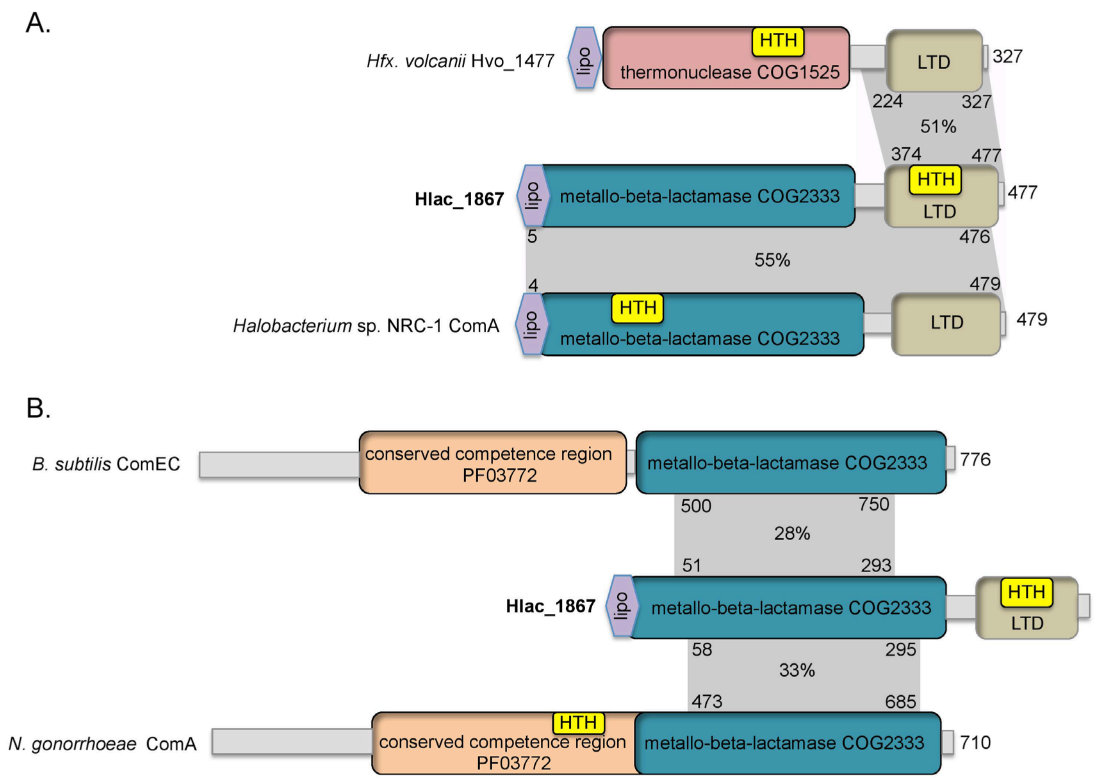
A.
Hfx. volcanii Hvo_1477
lipo
HTH
thermonuclease COG1525
LTD
327
224
327
51%
374
477
Hlac_1867
lipo
metallo-beta-lactamase COG2333
HTH
LTD
477
5
476
55%
4
479
Halobacterium sp. NRC-1 ComA
lipo
HTH
metallo-beta-lactamase COG2333
LTD
479
B.
B. subtilis ComEC
conserved competence region PF03772
metallo-beta-lactamase COG2333
776
500
750
28%
51
293
Hlac_1867
lipo
metallo-beta-lactamase COG2333
HTH
LTD
58
295
33%
473
685
N. gonorrhoeae ComA
HTH
conserved competence region PF03772
metallo-beta-lactamase COG2333
710

Figure S6. Statistical analyses of iTRAQ data from repeat experiments.
The proteins derived from the whole cell fraction in one labelling experiment that were differentially abundant (stationary vs log phase in medium B) were used for statistical analyses to assess reproducibility. The statistical analyses between biological replicates (A), technical replicates (B) and 8-plex iTRAQ labelling experiment replicates (C) were performed using SPSS 22.0. Unpaired data were removed. The linear relationship between biological replicates, technical replicates and experiment replicates was determined by examining the Analysis of Variance. The F-test outcome is highly statistically significant at a level of less than 0.001, suggesting a linear relationship among the variables. A: The Pearson correlation coefficient was 0.956 for the biological replicates and the correlation was significant at the 0.01 level (2-tailed). The regression equation for the biological replicates was $y=0.09+0.86x$ ($R^2=0.914$). B: The Pearson correlation coefficient was 0.825 for the technical replicates and the correlation was significant at the 0.01 level (2-tailed). The regression equation for the biological replicates was $y=0.19+0.86x$ ($R^2=0.681$). C: The Pearson correlation coefficient was 0.709 for the experiment replicates and the correlation was significant at the 0.01 level (2-tailed). The regression equation for the biological replicates was $y=-0.25+1.5x$ ($R^2=0.503$).

Figure S6

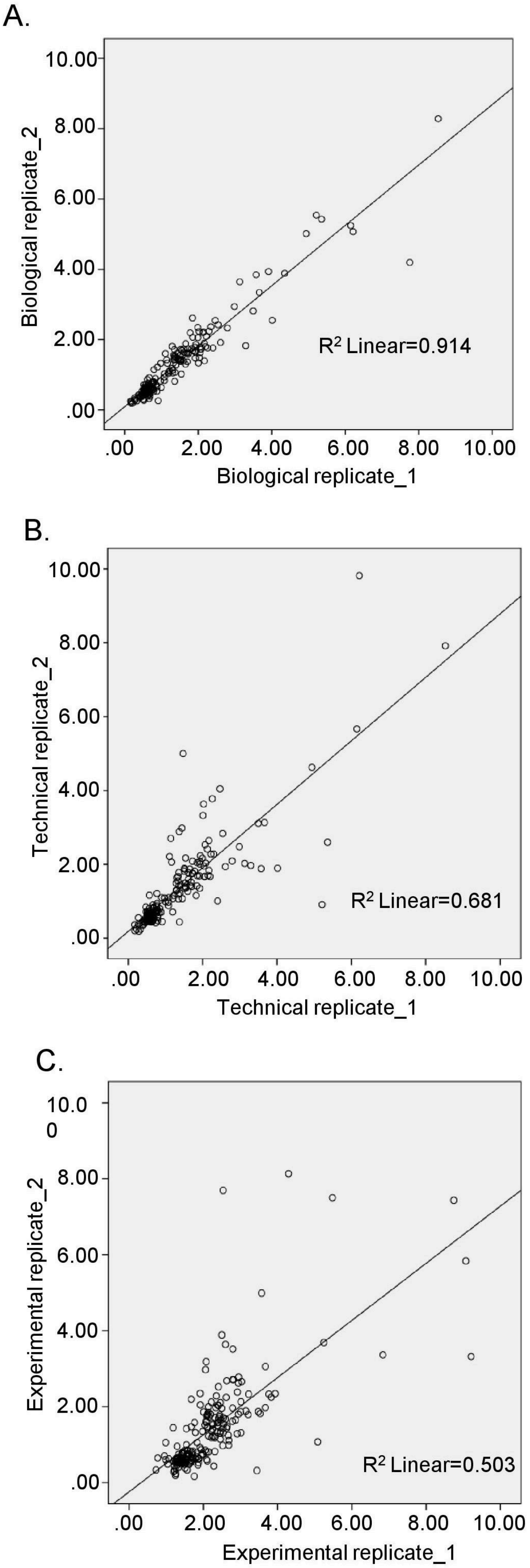
A.
10.00
8.00
6.00
4.00
2.00
.00
Biological replicate_2
R^2 Linear=0.914
.00
2.00
4.00
6.00
8.00
10.00
Biological replicate_1
B.
10.00
8.00
6.00
4.00
2.00
.00
Technical replicate_2
R^2 Linear=0.681
.00
2.00
4.00
6.00
8.00
10.00
Technical replicate_1
C.
10.0
0
8.00
6.00
4.00
2.00
.00
Experimental replicate_2
R^2 Linear=0.503
.00
2.00
4.00
6.00
8.00
10.00
Experimental replicate_1

Table S1. Labelling design for samples using the iTRAQ 8-plex format

iTRAQ reporter ion	Growth medium	Growth phase	Cell phenotype
113	Medium A	log	planktonic
114	Medium A	log	planktonic
115	Medium A	stationary	planktonic
116	Medium A	stationary	planktonic
117	Medium B	log	planktonic
118	Medium B	log	planktonic
119	Medium B	stationary	biofilm
121	Medium B	stationary	biofilm
